# Why Substantial Budget Reallocations Are Not and Should Not Be a Major Factor in Active Purchasing in Dutch Healthcare: Comment on "Measuring Active Purchasing in Healthcare: Analysing Reallocations of Funds Between Providers to Evaluate Purchasing Systems Performance in the Netherlands"

**DOI:** 10.34172/ijhpm.9115

**Published:** 2025-08-30

**Authors:** Fredo Schotanus

**Affiliations:** School of Economics, Utrecht University, Utrecht, The Netherlands.

**Keywords:** Managed Competition, Purchasing, Healthcare Reform, The Netherlands

## Abstract

This commentary discusses the study by Stadhouders et al, which analyzes budget reallocations among Dutch healthcare providers as a result of one form of active purchasing. The study assumes that healthcare purchasers aim to shift substantial funds from inefficient to efficient providers, yet finds little evidence of such shifts. This commentary explains more explicitly why substantial volume shifts are not and should not be a major factor in the Dutch context, citing factors such as the scarcity of underperforming providers, strong regional dependencies, data quality limitations, and patient reluctance to change provider. More promising avenues for active purchasing include fostering active collaboration and improving contractual arrangements.

## Introduction

 In their recent article, “Measuring Active Purchasing in Healthcare: Analyzing Reallocations of Funds Between Providers to Evaluate Purchasing Systems Performance in the Netherlands,” Stadhouders et al examine different healthcare purchasing systems in the Netherlands to assess how effectively these systems allocate funds across providers.^1^ To evaluate this, the authors develop a Market Activity Index to measure budget reallocations between providers.

 A key assumption in the article is that when healthcare purchasers engage in active purchasing, one of their aims would be to achieve “substantial reallocations of expenditures towards efficient providers.”^2^ This assumption raises the question whether substantial budget reallocations should, in fact, be a goal for Dutch healthcare purchasers. This commentary argues that such reallocations are, and should remain, relatively limited in the three markets where Stadhouders et al expect volume shifts. The reasons lie in factors such as scarcity of underperforming providers, limited provider capacity, strong regional dependencies, high switching costs, data quality limitations, patient reluctance to switch, and the importance of maintaining continuity of care, as discussed in the following sections.

## Constraints on Budget Reallocation for Hospital Care

 In the Dutch hospital care system, a limited number of health insurers contract providers. Insurers have a duty of care, meaning they must ensure adequate access to care. Dutch citizens are required to have health insurance and can switch insurers annually. Additionally, national agreements beyond insurer–provider contracting guide relocation of care, such as volume thresholds for complex care to improve quality.^3^ Finally, a housing shortage in the Netherlands makes relocation of healthcare staff challenging.

 In this context, several factors explain why substantial volume shifts in hospital care are unlikely and undesirable. First, there are generally no severely underperforming providers due to regulations, quality standards, and oversight.^4^ Consequently, substantial volume shifts in this regard would amount to unnecessary reshuffling, generating transaction costs without added value.

 Second, if insurers do decide to contract selectively to reallocate funds, they are likely to proceed cautiously, given that patients’ freedom to choose their healthcare provider is highly valued in the Netherlands.^5^ Insurers may also prefer to exclude providers where they already have limited business, while avoiding volume shifts that could jeopardize relationships with large hospitals on which they depend for capacity. Such shifts could also undermine established relationships at a time when collaboration across the healthcare system is increasingly prioritized.^6^ Moreover, insurers may need to ensure that providers with important regional functions are preserved. The bankruptcy of two hospitals in 2018, which sparked public outrage, illustrates how undesirable the loss of key providers can be perceived.^6^

 Third, if certain types of care are to be shifted from, for example, secondary to primary care, this will need to happen gradually to avoid overburdening primary care. At the same time, scaling down or reorganizing secondary care will also take time.

 Finally, while insurers could contract selectively, the annual option for Dutch individuals to switch insurers raises the question to what extent patients would actually change providers for planned care. If someone regularly uses a particular provider or wants treatment from a provider that is no longer contracted, they may switch insurers rather than providers. Research also shows that chronically ill and disabled individuals value the scope and coverage of an insurance more than the general population.^7^ From this perspective as well, volume shifts appear both ineffective and undesirable.

 In sum, contrary to the assumptions of Stadhouders et al, it is not expected that purchasers will pursue structural volume shifts as an important part of an active purchasing strategy. In fact, pursuing such shifts could even be counterproductive.

## Constraints on Budget Reallocation for Social Care

 Dutch social care is procured by municipalities, which have considerable discretion in how they organize it but are required to ensure accessibility and quality for eligible residents. Compared to insurers, municipalities operate under even greater political and societal pressure. They also need to consider the regional functions of providers in surrounding areas. Furthermore, during the examined period, most municipalities adopted a relatively passive purchasing approach, awarding contracts to all providers that met the requirements.^8^

 In this context, several factors—in addition to most of the factors mentioned for hospital care—explain why there are no structural volume shifts in social care and why this would be undesirable. First, a substantial share of the volume goes to a limited number of system providers on whom municipalities heavily rely, making large-scale adjustments difficult and risky. This difficulty stems from the fact that there is rarely an immediate alternative to the system providers. Even if a provider were to claim during a procurement process that it could take over a substantial volume, it is uncertain whether it could actually do so, as this would require considerable staff capacity, system adjustments, and other resources. More flexibility exists for shifting volume between smaller providers, but this would naturally no longer involve substantial volumes.

 Second, while municipalities face some pressure to use procurement effectively, there is also pressure to maintain a broad range of providers. In addition, selective purchasing can increase administrative burdens^8^ or even exclude capable providers, either because providers lack experience with tender procedures or because it can be genuinely difficult for a municipality to design an appropriate supplier selection model.^9,10^ As a result, many municipalities opt for procurement models that lead to broad contracting, either by directly contracting a wide range of providers or indirectly through contracting one or a few main contractors who, in turn, engage many subcontractors.

 Finally, effective volume shifting requires reliable data on care outcomes.^10^ For social care, this is even harder to measure than for hospital care. This stems from factors such as fewer standardized indicators for social care, limited national coordination, the difficulty of capturing outcomes like quality of life or self-reliance, and the influence of external factors beyond the care system. Making purchasing decisions without a clear view of outcomes is, understandably, undesirable.

 Although these factors are most evident in social care, they also apply—at least in part—to hospital care.

## Constraints on Budget Reallocation for Personal Budgets

 The personal budget in the Netherlands allows individuals who need care or support to purchase it themselves. This enables tailored care, particularly for those with complex needs, and often involves selecting providers from the budget holder’s own network, including family or friends.

 Similar to the other systems, several factors make significant volume shifts in this system both unlikely and undesirable. First, there is no central purchaser, such as a municipality or insurer, capable of actively shifting volumes on a large scale.

 Second, many personal budget holders employ family members, friends, or self-employed caregivers with whom recipients have long-standing and trusted relationships. Moving this volume from personal networks to professional providers would be costly, might not deliver clear quality improvements, and would run counter to the principle of encouraging budget holders to use their own networks.

 Finally, expecting personal budget holders to select providers efficiently through active purchasing seems unrealistic. Most do not have the capacity, procurement expertise, or market knowledge required. Asking budget holders to replace trusted relationships with new, unfamiliar providers also seems risky for them, could undermine care quality, and provoke resistance.

## Temporary Volume Shifts

 Across all markets analyzed, substantial volume shifts appear neither likely nor desirable. Still, the data from Stadhouders et al show one clear peak during a major reform in the Netherlands. While much of this reallocation seems to stem from administrative changes, there is some evidence of a temporary volume shift in social care and personal budget procurement.

 This is somewhat surprising, as several municipalities deliberately adopted procurement strategies after the reform to minimize change.^11^ In such cases, one would expect no, or only minimal volume shifts. Where municipalities did adopt new procurement strategies, shifts were more likely, either intentionally, for example to leverage informal care networks and reduce costs, or unintentionally, due to limited market knowledge as municipalities were inexperienced healthcare purchasers immediately after the reform. These effects proved temporary. Following the reforms, municipalities increasingly replaced short-term with long-term contracts in order to build more stable relationships with providers, create more room for service optimization, and avoid the inefficiencies of regularly changing procurement models.^12^

## Policy and Research Implications

 If substantial volume shifts are both rare and undesirable, the practical value of the Market Activity Index developed by Stadhouders et al is limited in countries such as the Netherlands. Its most relevant application may be to monitor whether new major reforms cause unintended reallocations. The index could, however, be more useful in countries with quality concerns or large differences in cost efficiency between providers.

 The implications and directions for future research in this commentary differ from those suggested by Stadhouders et al. In the Netherlands, policy-makers and healthcare purchasers could be better served by focusing on other forms of active purchasing. For example, purchasers could pay more attention to facilitating active collaboration between providers.^6^ Stronger collaboration also justifies longer-term contracts and targeted incentives, such as shared savings, innovation budgets, pilot projects, and sustainability targets.^13^ A longer-term, collaborative approach creates stability and predictability, enabling providers to implement innovations that require time to yield benefits, such as preventive care or the transition from disposable to reusable materials. Additionally, Dutch insurers could play a greater role in promoting value-based procurement within hospitals, where adoption is currently hindered by price pressures during negotiations with insurers and the prevalence of short-term contracts.^14^ This can encourage innovation and support sustainable care delivery by rewarding providers based on outcomes rather than volume.

 Future research could therefore move beyond selective contracting in systems like the Dutch one, as Stadhouders et al propose, and explore when and how alternative forms of active purchasing can be effective. This would build on strategic purchasing research across health systems, while research into active collaboration offers particular promise for rebuilding trust between the different actors in healthcare.^6^

## Ethical issues

 Not applicable.

## Conflicts of interest

 Author declares that he has no conflicts of interest.
